# Never Let a Crisis Go to Waste: Recruiting the Next Generation of Infectious Diseases Physicians

**DOI:** 10.20411/pai.v2i2.197

**Published:** 2017-06-27

**Authors:** Curtis J. Donskey

**Affiliations:** 1 Infectious Diseases Section, Cleveland Veterans Affairs Medical Center, Cleveland, Ohio; 2 Case Western Reserve University School of Medicine, Cleveland, Ohio

**Keywords:** infectious diseases, emerging infections, antibiotics, subspecialty, National Resident Matching Program, fellowship program

Sometimes infectious diseases can seem to be the least interesting subspecialty of internal medicine. We don't offer lifesaving or pain-relieving procedures. We offer the same advice over and over again hoping someday it might stick: wash your hands; stop prescribing antibiotics when there is no evidence of infection; and take your flu shot—it really won't give you the flu. We give advice that some consider expendable. Shouldn't all physicians be able to prescribe antibiotics with a little help from an online textbook or computer app? On top of all that, we apparently aren't very stylish. One of my female colleagues recently told me she can pick out all of the male infectious disease physicians at scientific meetings by the standard uniform of ill-fitting khakis and button-down shirts.

But a new crisis is always lurking to remind us that infectious diseases can be one of the most interesting and challenging areas of medicine. Emerging infections—often exotic and frightening— grab the attention of everyone from frontline personnel to the news media: Legionnaires' disease; HIV; West Nile virus; SARS; MERS; chikungunya; Ebola virus; Zika virus; and *Mycobacterium chimaera* to name just a few. Without warning, common pathogens create havoc when they acquire new resistance mechanisms or virulence factors: multidrug-resistant gram-negative bacilli; *Staphylococcus aureus*; and *Clostridium difficile*. Standard procedures such as transrectal biopsy of the prostate and endoscopic retrograde cholangiopancreatography suddenly become risky due to increasing antimicrobial resistance or inadequate methods for device reprocessing.

Former White House Chief of Staff Rahm Emanuel said of politics “never let a serious crisis go to waste.” This is also apt advice for infectious disease crises, particularly because tremendous preparation is often put into influxes of cases that never materialize. During the recent Ebola virus outbreak in Africa, infection control programs throughout the United States expended enormous amounts of time and money preparing, but only a handful of hospitals managed actual cases. Were these efforts wasted? No. Systems were put in place that will make us better prepared for future outbreaks. Scary reports of personnel acquiring Ebola virus despite the use of full-body coverage personal protective equipment stimulated efforts to improve the design of protective equipment and to train personnel to use it more effectively [1].

A recent crisis facing infectious diseases is not an exotic new pathogen. For the past several years, there has been a steady decline in the number of internal medicine residents applying for fellowship positions in infectious diseases. In 2015, more than half of infectious diseases training programs failed to fill all of their training positions in the National Resident Matching Program. Nephrology has seen similar declines. The crisis has led to a great deal of angst and soul-searching. Why is the field not attractive to new trainees? A number of challenges have been identified and some potential solutions have been proposed [2–3]. Leaders in the field have issued a call for all infectious diseases physicians to look for opportunities to mentor medical students and residents and increase their exposure to the many positive aspects of the field.

All of this discussion caused me to think back on my own decision to go into the field of infectious diseases. The decision didn't have anything to do with the things that make my current job exciting—emerging infections, outbreaks, infection control disasters. As a medical resident, I found these were interesting facts to be memorized for tests, but they seemed far removed from routine patient care. My decision occurred during the course of an infectious diseases elective when I was a second-year resident in internal medicine. I entered the elective with no thought of choosing infectious diseases as a career—I just wanted to learn how to choose antibiotics. My opinion began to change with the first patient I saw on the consult service.

I only had a few minutes to interview the patient. I saw him between tests being completed in preparation for surgery. He was scheduled for amputation of his leg just below the knee because an infected diabetic foot ulcer had not responded to first oral and then intravenous antibiotics. A large amount of pus had spontaneously drained five days earlier, leaving an open draining ulcer. He was frustrated, but resigned to the need for surgery. The question for the consult service seemed simple: What antibiotics and for how long?

Dr. John Boyce, the attending on the Infectious Diseases consult service, asked more questions than I had anticipated. Did the presentation of the illness seem odd? The ulcer was on the top of the foot proximal to the first two toes; most diabetic foot ulcers are on the sole of the foot where pressure is applied when walking. Had there been some trauma or pressure to the area from ill-fitting shoes? Why were the gram stain and cultures of the pus from the foot negative? If the lack of improvement meant the antibiotics weren't working, why hadn't an antibiotic-resistant organism grown in the culture?

After carefully examining the foot, Dr. Boyce asked the patient “Have you ever had gout?” Yes, he replied, but he couldn't remember the details. After sorting through a stack of medical records, we found confirmation of the diagnosis three years earlier. The consult team delivered a swab from the patient's foot to the lab and viewed the telltale uric acid crystals with the pathologist. The surgery was cancelled. The patient and his wife were overjoyed. Dr. Boyce left a note in the chart using his trademark green pen and finished rounds with a slideshow on various noninfectious conditions including gout that mimicked infection and were unfortunately not diagnosed until review of pathologic findings after amputation.

Any physician can prescribe antibiotics. If anything, it is too easy. A new generation of well-trained infectious diseases physicians is needed to tackle the hard work involved in making correct diagnoses, choosing antimicrobials wisely, and stopping them as soon as possible. And just when that work starts to seem tedious, they need to be ready to deal with the new crises that are sure to come up.

In addressing the dearth of interest in infectious diseases, it may be useful to ask infectious disease physicians to think back on why they entered the field and to contemplate why their current positions are or are not satisfying. Surveys are useful. Some good stories might also be helpful. The late Emanuel Wolinsky survived a stay in a tuberculosis sanatorium and went on to devote a large part of his career to the care of patients with mycobacterial infections [4]. Others have devoted their careers to fighting the HIV epidemic or the continuing emergence of antibiotic-resistant pathogens. Someone can also tell the story of how they considered choosing infectious diseases, but chose another subspecialty because they had loans to repay. So, let's start writing. If we can gain insights into how to inspire new recruits, the crisis will not have been wasted. And if this fails, we always have the fallback plan of upgrading our wardrobes.

## References

[B1] Donskey C Never Let a Crisis Go to Waste: Recruiting the Next Generation of Infectious Diseases Physicians. Pathogens and Immunity. 2017;2(2):270–3. doi: 10.20411/pai.v2i2.197 28819651 PMC5557299

